# In Situ Self-Assembled Nanocomposites from Bacterial Cellulose Reinforced with Eletrospun Poly(lactic acid)/Lipids Nanofibers

**DOI:** 10.3390/polym9050179

**Published:** 2017-05-19

**Authors:** Chunhui Xiang, Nuria C. Acevedo

**Affiliations:** 1 Department of Apparel, Events, and Hospitality Management, Iowa State University, Ames, IA 50011, USA; 2 Department of Food Science and Human Nutrition, Iowa State University, Ames, IA 50011, USA

**Keywords:** bacterial cellulose nanocomposites, in situ self-assembly, hydrophobicity

## Abstract

The goal of this study is to explore a new strategy to improve the mechanical and hydrophobic properties of bacterial cellulose (BC) mats. The present work is the first to report the preparation of in situ self-assembled BC nanocomposites using electrospun hydrophobic poly(lactic acid) (PLA) or PLA/lipids (PLA/Lip) nanofiber mats as foundation for BC nanofiber growth. Adding electrospun PLA mats to the BC culture media led to a two-fold increase in toughness with a 52% increase in elongation of the nanocomposites with regard to BC. The incorporation of electrospun PLA and PLA/Lip nanofiber mats lowered the moisture regain and water vapor transmission of BC nanocomposites relative to pure BC mats. The interfacial bonding between the individual components of a nanocomposite is a key factor for the improvement of composite strength, stiffness, and barrier properties; thus additional strategies to improve interaction between hydrophilic BC and hydrophobic PLA fibers need to be explored.

## 1. Introduction

The development of sustainable natural-based biopolymer to replace petrochemical-based materials has attracted much attention over the last decade [1,2,3,4]. Bacterial cellulose (BC) is a biodegradable polymer produced by Acetobacter bacteria through a hierarchical cell-directed self-assembly process. BC is characterized by having the same chemical structure (a polysaccharide consisting of a linear chain of β(1 → 4) linked d-glucose units) as the plant-based cellulose [2]. BC displays excellent mechanical properties due to the ultrafine-fiber network structure, the good chemical stability and the high water absorption capacity [5]. In addition, its thermal properties, biodegradability, and biocompatibility make BC a promising material for different end uses. Many methods have been used to fabricate BC for different applications including textiles, nanocomposite membranes, foods, etc. [6].

BC nanocomposites can be produced by (1) blending BC with a second phase after the nanofiber network structure is formed; in this case, BC can be used as a direct matrix reinforced with polymeric or mineral fillers, or it can be the added as a reinforcement to another polymer [5]; and/or (2) adding a second phase into the culture media during the nanofiber matrix formation in order to create a double network structure [5,7,8,9]. A variety of methods (such as electrospinning, in situ self-assembly, particle deposition, dispersion, and casting) have been utilized for achieving improvement of the physical and functional properties of BC nanocomposites [7]. Both Park et al. [10] and Martinez-Sanz et al. [11] reported using BC whiskers/nanowhiskers as reinforcement to electrospun nanofibers. BC whiskers/nanowhiskers were incorporated into solutions before fiber electrospinning.

Electrospun mats of fibers with nanoscale diameters can act as sponges to absorb chemicals based on both the small pore size formed between the fibers and the large surface area of the fibers available for sorption phenomena [12]. PLA has been extensively studied when produced by electrospinning for various applications [13,14]. Nevertheless, there is no literature research reporting on in situ self-assembled nanocomposites of BC reinforced with electrospun hydrophobic PLA nanofiber mats. By taking advantage of the large surface area-volume ratio and high porous structure of PLA nanofibers, which favors the capillary action (wicking) will lead to the growing of BC nanofibers among the pores of the electrospun PLA nanofiber mats.

The present work is the first to report the production of in situ self-assembled BC nanocomposites using electrospun hydrophobic PLA or PLA/lipids (PLA/Lip) nanofiber mats as foundation for BC nanofiber growth as a strategy to improve the mechanical properties and to decrease water absorption of BC. In an effort to increase BC nanofiber hydrophobicity as well as to improve the interface between the PLA matrix and BC fibers, lipids were dissolved into PLA/chloroform–acetone solution before electrospinning. The incorporation of lipids to films has been previously used to reduce water sorption and water transfer through film, and hence to increase the hydrophobicity of films reducing the water vapor permeability [15,16].

The mechanical properties, structure, thermal behavior, viscoelastic properties, and hydrophobicity of the BC nanocomposites were characterized. It is expected that the nanocomposite of BC and PLA will present better mechanical properties and lower water absorption compared to the pure BC mats. New products developed from the resulting nanocomposites will be sustainable, have good tensile strength, and relatively low moisture regain, which are the key parameters for the materials used in textiles for regular daily wear.

## 2. Materials and Methods

### 2.1. Materials

Poly(l,l-lactide) (PLA 4032D) in pellets containing 1.4% d-isomer, and average molecular weight of 220 kDa, was purchased from NatureWorks LLC (Wayzata, MN, USA). SCOBY (symbiotic colony of bacteria and yeast) was purchased from Kombucha Kamp (Hatfield, MA, USA). Lipids (Stearic acid) were purchased from Sigma-Aldrich (St. Louis, MO, USA). Chloroform and acetone were obtained from Fisher Scientific (Hampton, NH, USA). All reagents were used without further purification.

### 2.2. Preparation of Bacterial Cellulose

Commercially available SCOBY (including Acetobacter bacterial species, as well as various Saccharomyces and other yeast types) was cultured in a static medium (initial pH = 5.0, room temperature) in 190 mm dia. × 100 mm H crystallizing dishes containing 500 mL medium. The standard formula of the culture media was: 3760 mL distilled water, 540 granulated cane sugar, 632 mL white vinegar (5% acidity), and approximately 100 g commercially available SCOBY. The bacterial cellulose formed on the air-medium interface and the samples were harvested five weeks after cultivation.

### 2.3. Preparation of BC/PLA and BC/PLA/Lip Nanocomposites

PLA nanofibers were electrospun from 8 wt % PLA dissolved in chloroform/acetone (3:1, *v*/*v*). PLA/Lip nanofibers were electrospun from 8 wt % PLA in chloroform/acetone (3:1, *v*/*v*) containing 5% lipids (PLA *w*/*w*). The lipid used in this study was stearic acid. During electrospinning, the polymer solution was introduced into a 10 mL glass syringe (VWR Scientific, West Chester, PA, USA). The syringe was attached to a metal needle (ID = 0.60 mm). Electrospinning was performed at 15 kV by a high voltage supply (Gamma High Voltage Research Inc., Ormond Beach, FL, USA) and at 10 µL/min feed rate driven by a programmable syringe micropump (Harvard Apparatus, Holliston, MA, USA). A rotating aluminum drum (diameter = 10 cm) covered with aluminum foil was used to collect PLA and PLA/Lip fiber mats at a 15 cm distance away from the needle tip. Each sample was collected for 2 h. The electrospun PLA and PLA/Lip nanofiber mats were placed on the surface of the culture medium at the beginning of the inoculation to allow the growth of BC fibers in between the open spaces or pores of the PLA or PLA/lip mats. The size of the PLA and/or PLA/Lip mats was the same as the surface area of the culture media. The BC mats formed on the air-medium interface and the nanocomposite samples were harvested five weeks after cultivation. To ensure homogeneity of the mats obtained, the central section (100 mm diameter) was used for analysis. Three types of nanocomposites were obtained: (1) pure bacterial cellulose (BC); (2) BC/PLA nanocomposite; and (3) BC/PLA/Lip nanocomposites.

### 2.4. Characterization of the Nanocomposite Materials

#### 2.4.1. Scanning Electron Microscopy (SEM)

Surface morphology of the nanocomposites was examined using SEM and Quanta-250 Field Emission Scanning Electron Microscopy (FESEM, FEI Company, Hillsboro, OR, USA). All samples were mounted on 1″ stubs and coated with a 5 nm layer of gold/palladium to increase conductivity and prevent any charging during the imaging process. Image J 1.42q (Bethesda, MD, USA) was employed to evaluate the fiber diameter of the nanocomposites.

#### 2.4.2. Fourier Transform Infrared Spectroscopy (FTIR)

The BC, BC/PLA, and BC/PLA/Lip nanocomposites were characterized using an FTIR spectrophotometer (Bruker IFS-66v FT-IR, Billerica, MA, USA). FTIR spectra were taken in the range of 4000–800 cm^−1^ wavenumbers using a split pea accessory. Each scan was an average of 64 scans obtained at a resolution of 4 cm^−1^.

#### 2.4.3. Differential Scanning Calorimetry (DSC)

Thermal analysis of the different nanocomposites was carried out with a DSC Q2000 (TA Instruments, New Castle, DE, USA). The instrument heat capacity response was calibrated with sapphire, and the heat flow was calibrated with indium. Approximately 5 mg of the samples were placed in alodined pans and sealed hermetically (an empty pan served as reference). Samples were heated from 10 to 200 °C with a ramp of 10 °C/min. Thermograms were evaluated using TA Universal Analysis 2000 Software (TA Instruments, New Castle, DE, USA). The glass transition (*T*_g_), peak melting temperature (*T*_m_) and the enthalpy of melting (Δ*H*_m_) were determined. The averages and standard deviations of four replicates are reported in this study.

#### 2.4.4. X-ray Diffraction (XRD) Analysis

X-ray diffraction patterns of the nanocomposites were obtained using a Rigaku Multiflex Powder X-ray Diffractometer (Rigaku, Japan). The copper lamp (λ = 1.54) was set to 40 kV and 44 mA. The samples were scanned from 5 to 40 degrees at 1°/min and the patterns analyzed with MDI’s Jade 6.5 software (Rigaku, Japan). Results of at least three replicates are reported.

The relative crystallinity index was calculated by the Segal’s method [17], through the equation

(1) $$CI=\frac{\left[I\left(002\right)-I_{am}\right]}{I\left(002\right)}\;100$$

where *I*(002) is the peak intensity of the (002) lattice diffraction at 2*q* = 22.9° for cellulose *I*, and *I_am_* is the intensity diffraction of the amorphous fraction at 2*q* = 18°.

#### 2.4.5. Tensile Tests

Tensile properties of the BC, BC/PLA, and BC/PLA/Lip nanocomposites were determined using an Instron Universal Testing machine (model 4502, Universal Testing Systems, Norwood, MA, USA) following the guidelines of ASTM D638-10. Nanocomposites were cut into dog-bone shaped samples. These dog-bone shaped samples had the following dimensions: length 75 mm, gauge length 30 mm, thickness 0.2 mm; the narrowest part of the sample was 4 mm. Tensile test were based on the test conditions using a 10 kN load cell and a strain rate of 10 mm/min. All samples were tested for at least five replicate measurements to obtain reliable data. Mean values and standard deviations are reported.

#### 2.4.6. Moisture Absorption Characteristics

Samples (25 mm × 25 mm, 0.9 mm thickness) were cut from BC, BC/PLA, and BC/PLA/Lip nanocomposites. All samples were bone-dried in a vacuum oven at 40 °C for 24 h. After drying, the samples were weighed and allowed to absorb moisture in an environment of 65 ± 2% RH at 21 ± 0.5 °C. Samples were weighed every 24 h until no weight change was observed. During the experiment, the change in weight was monitored over time. Three specimens for each sample were examined, the average and standard deviation values were reported. Moisture regain, *M*, was calculated as

*M* = [(*w*_1_ − *w*_0_)/*w*_0_] × 100 (%) (2)

where *w*_0_ is the initial weight of sample (bone-dried) and *w*_1_ is the sample weight after absorbing moisture.

#### 2.4.7. Water Vapor Transmission Rate (WVTR) Analysis

WVTR was determined gravimetrically using a modification of ASTM E 96-95 (1995b). Briefly, a Petri dish was filled with distilled water, covered, and sealed with a film. The mass of water lost from the dish was monitored by the difference in weight as a function of time when placed in a convection oven at 37.0 ± 0.5 °C. WVTR was calculated using

(3) $$WVTR\;g/m^{2}h=\frac{mass\;of\;water\;lost}{time\;\times area}=\;\frac{flux\;}{area}$$

#### 2.4.8. Statistical Analysis

GraphPad Prism 5 software (GraphPad Software, Inc., San Diego, CA, USA) and OriginPro 9.1 software (OriginLab Corporation, Northampton, MA, USA) were used for data statistical analysis. Means and standard deviations are reported, and samples were statistically compared by using one-way ANOVA (*p* < 0.05) with Tukey’s multiple comparisons as a post-test (*p* < 0.05).

## 3. Results

### 3.1. SEM Analysis

**Figure 1:**
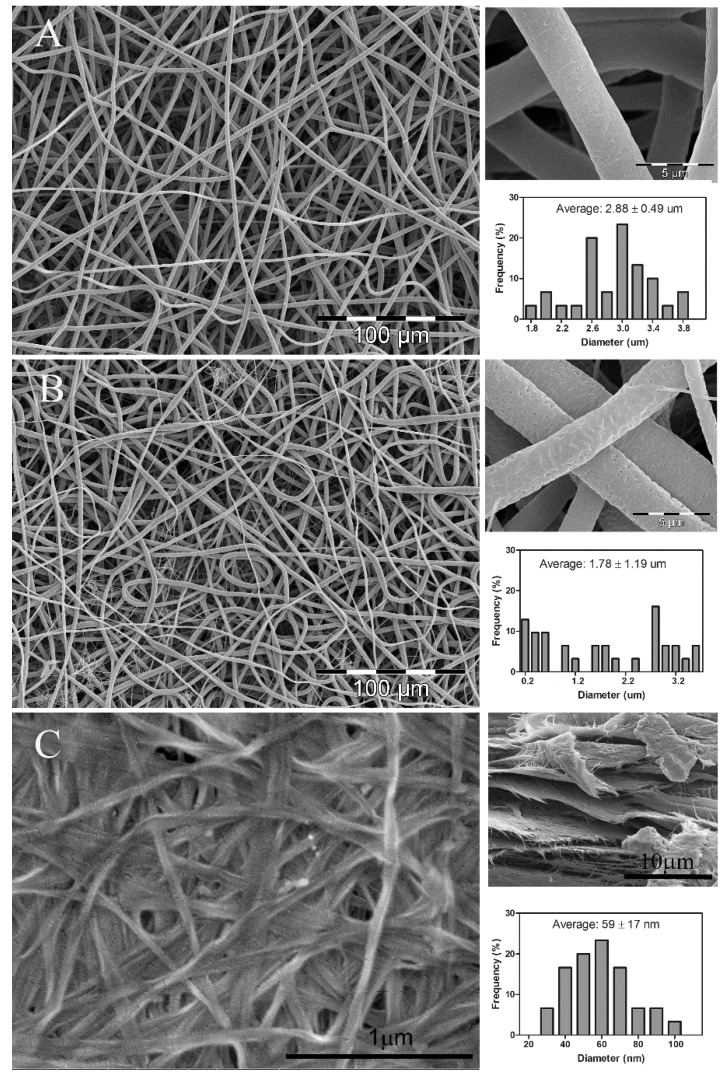
SEM images and fiber diameter distribution of nanofibers. (**A**) PLA from 8 wt % PLA in chloroform/acetone (3:1, *v*/*v*) solution; (**B**) PLA/Lip from 8 wt % PLA in chloroform/acetone (3:1, *v*/*v*) containing 5% lipids (PLA *w*/*w*); (**C**) BC sample surface.

Figure 1 shows the morphology and average fiber diameter of PLA nanofibers (Figure 1A), PLA/Lip (Figure 1B) and bacterial cellulose (BC) (Figure 1C). As expected, both PLA nanofibers (Figure 1A upper right) and PLA/Lip nanofibers (Figure 1B upper right) showed a porous structure attributed to the chloroform/acetone solvent system used for electrospinning [12]. The highly porous structured fibers are expecting to favor the capillary action (wicking) which will lead to the growing of BC nanofibers among the pores of the electrospun PLA nanofiber mats. The BC sample shows a highly fibrous network-like structure consisting of ultrafine nanofibrils (Figure 1C). The average fiber diameter of electrospun PLA fibers is 2.88 ± 0.49 μm with a normal distribution (Figure 1A lower right). While the electrospun PLA/Lip fibers have an average fiber diameter of 1.78 ± 1.19 μm with a random distribution (Figure 1B lower right). At the 0.05 significance level, the incorporation of lipids to the PLA/chloroform-acetone solution significantly decreased the average fiber diameter of the electrospun PLA fibers. This can be explained by the formation of nano-nets which is more significant in the presence of lipids. The nano-fiber/nets structure was also reported by other researchers [18,19,20]. Ding et al. [19] stated that the nano-nets are the result of the occurrence of phase separation of charged droplets generated during electrospinning. The average fiber diameter of the BC nanofibers is 59 ± 17 nm. These results are in good agreement with the work previously reported by Feng et al. [21]. A dense network of BC fibers was observed, as described by Bielecki et al. [22]. Bacterial cellulose mats are comprised of multiple layers of fiber networks of about 1 μm thick. It is also worth mentioning that no additional PLA-fiber morphological changes were observed as a result of the media conditions during the period of time this study took place. Thus, changes in porosity or fiber degradation can be considered negligible.

**Figure 2:**
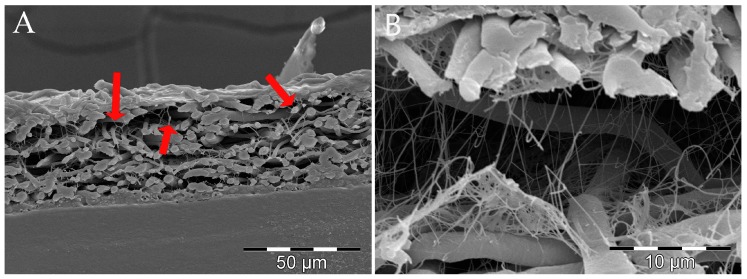
SEM images showing two different magnifications of the cross-section of BC/PLA nanocomposites. (**A**) The arrows indicate the presence of fibers along the thickness of the PLA mats. (**B**) Higher magnification image of BC/PLA/Lip nanocomposite.

The cross-section morphology of the BC/PLA nanocomposites is shown in Figure 2. It can be observed in Figure 2A) that the cellulose nanofibers grew in the open spaces along the whole thickness of electrospun PLA mats. BC nanofibers are present in the vicinity of the interface with air which indicates a significant penetration of BC fibers into the PLA mats occurred. The entanglement of PLA and BC nanofibers probably favored the structural utility of the nanocomposite. A similar entanglement of the PLA/Lip electrospun nanofibers and BC nanofibers was observed (Figure 2B) indicating that the BC nanofibers grew similarly along the pores of both electrospun PLA and PLA/Lip nanofiber mats.

### 3.2. FTIR Analysis

**Figure 3:**
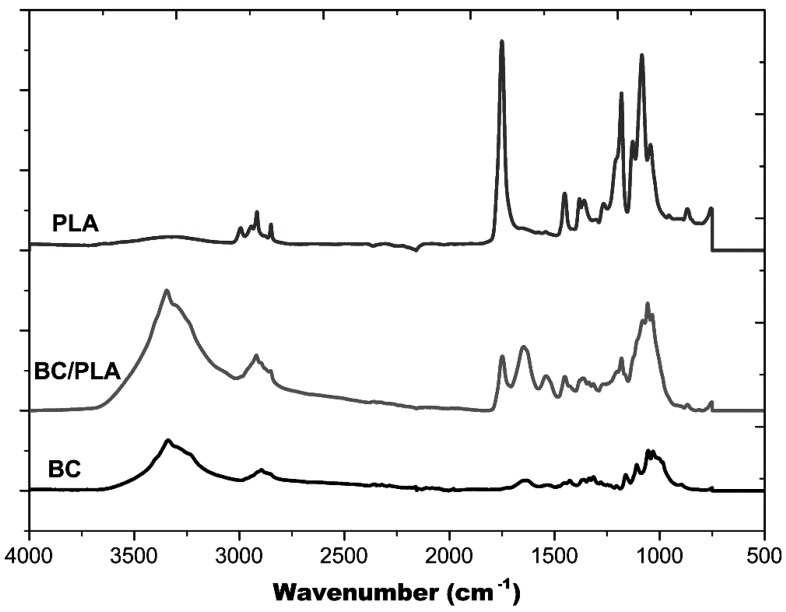
FTIR spectra of (top to bottom): pure BC, BC/PLA nanocomposite, and pure PLA.

Figure 3 shows the FTIR spectra of the BC mat, BC/PLA nanocomposite, and electrospun PLA fiber mat. A broad band at 3200–3500 cm^−1^ seen in BC and BC/PLA spectra, is the result of the hydroxyl (O–H) stretching vibration resulting from the strong intra- and inter-molecular hydrogen bonds [23]. The FTIR spectrum of PLA has an absorption peak appearing around 1746 cm^−1^, corresponding the carbonyl group of the branched PLA [24]. It can be observed that for BC/PLA nanocomposites, there is presence of the PLA absorption peak around 1746 cm^−1^ which appears in pure PLA, and the O–H stretching modes in alcoholic groups from pure BC. These results indicate the entangled structure of BC nanofibers and the electrospun PLA fibers and are consistent with the BC/PLA nanocomposite entanglement structure shown in Figure 2A.

### 3.3. X-ray Diffraction Analysis

**Figure 4:**
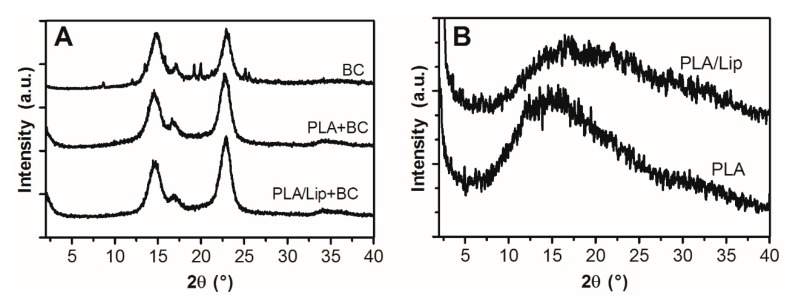
X-ray diffractograms of BC, BC/PLA, and BC/PLA/Lip nanocomposites (**A**), and electrospun PLA with and without lipids (**B**).

The XRD patterns of BC and BC/PLA with and without lipids are shown in Figure 4A. In the case of pure BC a typical pattern of cellulose I allomorph can be observed with four peaks centered at 2θ 14.7°, 17.0°, 22.9°, and 34.6°, corresponding to the diffraction planes 101, 10ī , 002 and 040, respectively [25,26]. No significant differences were observed in the BC X-ray patterns when PLA or PLA/Lip were incorporated, indicating that they did not affect the crystalline structure of the BC matrix.

We investigated the influence of PLA and PLA/Lip in the BC by mean of the calculation of nanocomposites crystallinity. The estimated relative crystallinity index (CI) of pure BC is 75.5% ± 2.5% which is in close agreement with previous works on BC [3,21,27,28]. Interestingly, significantly higher CI values were obtained for BC/PLA and BC/PLA/Lip (83.8% ± 4.3% and 83.1% ± 8%, respectively) which can be attributed to the ability of PLA fibers to act as a seed and favor crystallinity of the growing BC. Many extraneous factors can impact the micro-assembly of BC fibrils. It has been previously reported that during BC crystallization, cellulose molecules organize and stack together through hydrophobic interaction to form molecular sheets; then these sheets associate to each other by hydrogen bonds to form a crystal [29]. Due to their hydrophobic nature, it is possible that electrospun PLA fibers become somewhat adsorbed into the BC matrix, promoting hydrophobic stacking which eventually leads to a higher crystallinity compared to pure BC. However, further studies need to be performed to confirm this hypothesis. No additional peaks were observed in the BC/PLA and BC/PLA/Lip composite X-ray patterns, which can be explained by the low proportion of PLA and PLA/Lip present in the mats unable to be detected by XRD. On the other hand, an amorphous profile with high noise/signal ratio was observed for PLA and PLA/Lip fibers (Figure 4B). It has been previously reported that wide-angle X-ray diffraction profiles of bulk PLA membranes show a semicrystalline structure with two crystalline peaks at 2θ = 16.8° and 19.2° [30,31]. However, Gomez–Pachon et al. [30] reported that electrospinning can delay the crystallization process of PLA leading to less crystallinity of the polymers. This low crystallinity can be attributed to the rapid solidification of the stretched chains that occurs during electrospinning, preventing the development of a well-organized structure. As a result, the nanofibers are randomly oriented and therefore no evidence of extensive crystallization is found as observed in Figure 4B.

### 3.4. Differential Scanning Calorimetry

**Figure 5:**
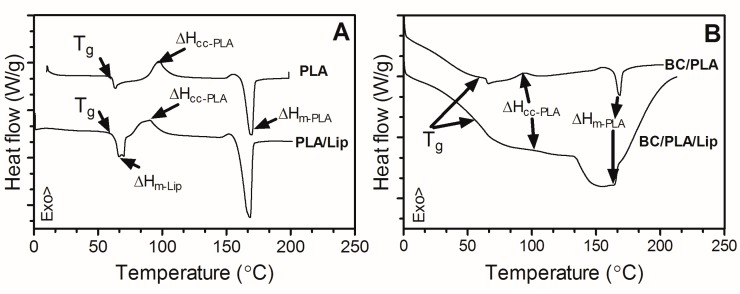
DSC plots of PLA (**A**) and BC/PLA (**B**) nanocomposites with and without incorporation of stearic acid.

Thermograms of neat PLA and BC/PLA nanocomposites before and after incorporation of lipids are shown in Figure 5. The observed glass transition (*T*_g_) and melting (*T*_m_) temperatures of electrospun PLA are in line to those reported by other authors [30,32,33]. Furthermore, an enthalpic endothermic relaxation can be observed at around 63 °C due to the rapid solidification rate during electrospinning, followed by a cold crystallization exothermic event at 94 °C. These results are in agreement with the X-ray patterns (Figure 4B) where it is evident the amorphous structure of the PLA samples. *T*_g_ of PLA was not significantly affected by the presence of lipids; however, it was found it influenced PLA’s crystallization behavior. PLA/Lip cold crystallization temperature (*T*_c_) showed a significant decrease (*p* < 0.05) when compared to pure PLA (Table 1) which suggests lipids promote PLA crystallization by favoring the molecular arrangement of PLA chains. These results are in line with the higher meting enthalpy (Δ*H*_m_) observed in PLA/Lip mats. Interestingly, the crystallization behavior of BC/PLA and BC/PLA/Lip showed different trends from those of PLA mats (Figure 5B). The heating scan of BC/PLA exhibits a *T*_c_ at about 89 °C, a value significantly smaller (*p* < 0.05) than that observed for pure PLA (Table 1). This behavior suggests a higher ability of PLA to crystallize in the biocomposite and can be associated, to BC nanofibers acting as nucleating agent. Furthermore, BC did not significantly influence *T*_g_ or *T*_m_ of PLA. Findings in line with these results can be found in the literature [34,35,36].

### 3.5. Mechanical Properties

**Figure 6:**
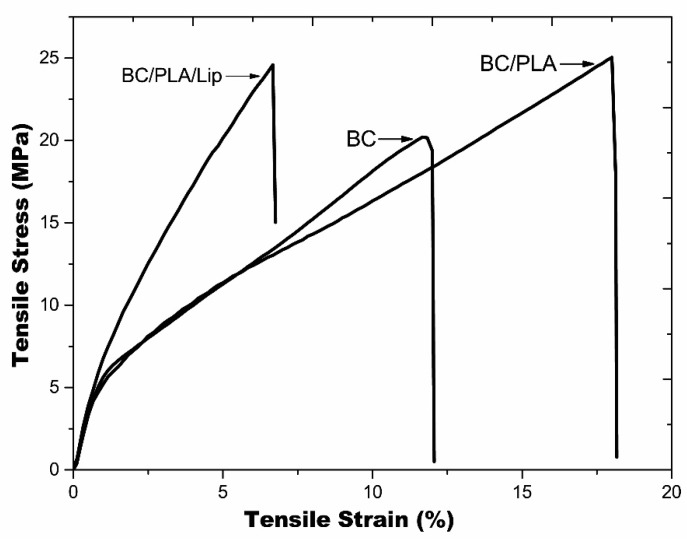
Stress versus Strain plots for BC, BC/PLA, and BC/PLA/Lip nanocomposites.

Figure 6 shows typical stress versus strain plots of BC, BC/PLA, and BC/PLA/Lip nanocomposites. As it is confirmed by the values presented in Table 2, the BC/PLA and BC/PLA/Lip nanocomposites showed no significant (*p* < 0.05) change in the tensile strength with regard to pure BC mats. The Young’s modulus of the BC mats was 575.5 MPa, while those of the BC/PLA and BC/PLA/Lip nanocomposites were 319.9 and 810.4 MPa (Table 2), respectively. Compared with pure BC mats, the toughness and elongation of the BC/PLA nanocomposites doubled and increased 52%, respectively (Table 2). This toughening effect was also reported by other researchers [37,38]. The elongation at break value of pure BC was approximately 12.3% whereas that of BC/PLA was 18.7%. Overall, an improvement of the mechanical properties was observed for the developed BC/PLA nanocomposites when compared with BC mats. These findings can be explained by the increased crystallinity of BC/PLA nanocomposites due to the presence of electrospun PLA. On the other hand, inferior mechanical properties were obtained for BC/PLA/Lip nanocomposites. For instance, a decrease of approximately 38% was observed in both elongation at break and toughness values in the BC/PLA/Lip when compared to BC/PLA. The negative impact of lipids on film mechanical properties was also reported by Péroval et al. [39].

### 3.6. Moisture Regain

**Figure 7:**
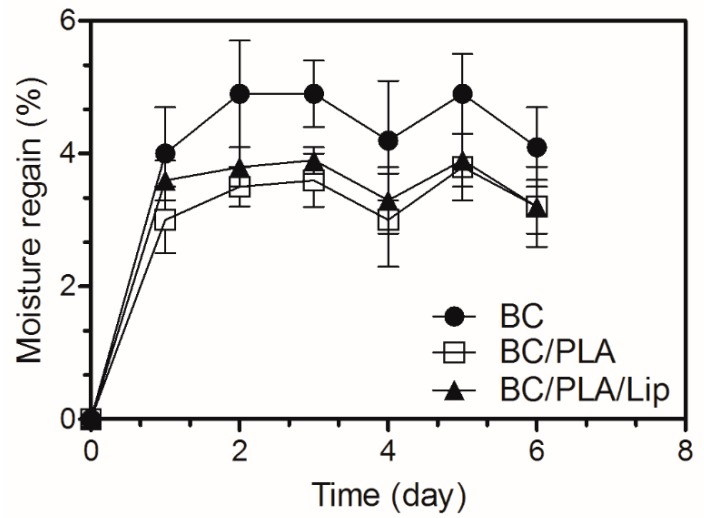
Moisture regain of BC, BC/PLA, and BC/PLA/Lip mats.

Cellulose-based nanocomposites are known to have poor moisture barrier properties due to their hydrophilic nature. Water sensitivity is a crucial aspect of cellulose-based nanocomposites because the moisture picked up upon immersion in water or in high humidity environments is detrimental to their mechanical strength and dimensional stability [40]. Moisture regain during absorption is shown in Figure 7 for BC, BC/PLA, and BC/PLA/Lip nanocomposites. The incorporation of electrospun PLA and PLA/Lip to the BC culture medium significantly decreased (*p* < 0.05) the moisture regain of the nanocomposites. While the moisture regain of BC/PLA and BC/PLA/Lip nanocomposites were not significantly different at the 0.05 level.

### 3.7. Water Barrier Properties

**Figure 8:**
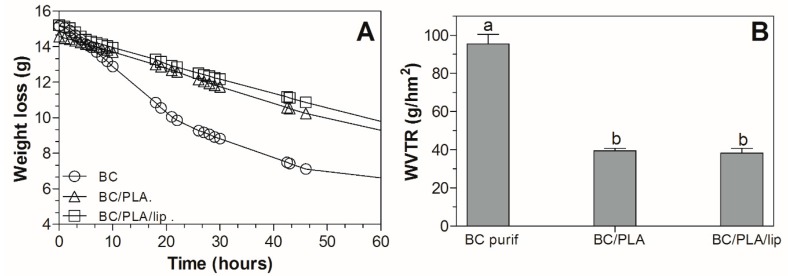
Weight loss as a function of time (**A**), WVTR values (g/hm^2^) of the nanocomposites (**B**).

The weight loss over time curves and water vapor transmission rate (WVTR) coefficients of the nanocomposites are shown in Figure 8. The growth of BC using electrospun PLA fibers as support significantly impacted the water transmission properties. Cellulose has a hydrophilic nature, therefore an improvement in the water barrier properties of the BC/PLA nanocomposites when compared to BC was expected. As observed in Figure 8A, the amount of water lost from the dish over time was more significant in BC mats and it is characterized by an initial sharp decrease in weight followed by a plateau reached approximately at 50 h incubation when ~50% of water was transferred from the dish through the BC mat. The presence of PLA fibers in the BC film decreased the WVTR value of the BC/PLA mats from 95.3 to 39.4 g/hm^2^ (Figure 8B). In the present work, electrospun PLA fibers not only improved the water vapor barrier, but also modified the nanocomposite surface as could be observed in the SEM micrographs (Figure 1A). There is a lack of literature reporting on the water barrier properties of BC/PLA nanocomposites; however, other researchers observed reductions in water permeability in PLA reinforced with cellulose nanowiskers using the casting method [41] and electrospinning-melt compounding [42]. Furthermore, Martínez-Sanz et al. [43] also reported and improvement of the water barrier properties when working with BC nanowisker films coated by electrospun-annealed PLA fibers. It is clear that the combination of hydrophilic BC and hydrophobic PLA is a feasible route to protect the nanocomposites from moisture. Surprisingly, among the BC/PLA nanocomposites, the WVTR did not significantly change with the addition of 5% lipids.

## 4. Conclusions

The results of this work show that the incorporation of electrospun PLA mats to the BC culture media led to an overall increase of the mechanical properties of the nanocomposites. X-ray diffraction measurements showed that PLA increased the crystallinity of BC/PLA nanocompities. This led to an intensification of the nanocomposites’ toughness compared to pure BC. The incorporation of lipids to the PLA solution before electrospinning was not suitable to improve nanocomposite strength. In fact, the addition of lipids led to a highly brittle and fragile material without any improvement of water permeability nor water regain. The importance of the interfacial bonding between the individual components is clear; thus, additional strategies to improve interaction between hydrophilic BC and hydrophobic PLA fibers need to be explored in order to improve composite strength, stiffness, and hydrophobicity.

## Figures and Tables

**Table 1:** Thermal properties of electrospun PLA and BC/PLA nanocomposites with and without incorporation of lipids.

	*T*_g_ (°C)	*T*_c_ (°C)	Δ*H*_c_ (°C)	*T*_m_ (°C)	Δ*H*_m_ (°C)
**PLA**	61.7 ± 0.4 ^a^	94.0 ±3.2 ^b^	21.2± 2.0 ^e^	168.7 ± 1.3 ^h^	38.8 ± 0.2 ^i^
**PLA/Lip**	63.5 ± 0.6 ^a^	88.7 ± 0.5 ^c^	22.3 ± 1.2 ^e^	167.8 ± 0.3 ^h^	46.9 ± 1.5 ^j^
**BC/PLA**	61.8 ± 1.2 ^a^	88.6 ±1.62 ^c^	1.9 ± 0.4 ^f^	166.5 ± 4.0 ^h^	5.4 ± 0.9 ^k^
**BC/PLA/Lip**	63.7 ± 2.6 ^a^	103.8 ± 1.4 ^d^	0.2 ± 0.1 ^g^	165.9 ± 0.5 ^h^	3.0 ± 1.9 ^l^

Different letters represents significantly different values (*p* < 0.05).

**Table 2:** Mechanical properties of BC, BC/PLA, and BC/PLA/Lip nanocomposites.

Samples	Young’s Modulus (MPa)	Tensile Strength (MPa)	Elongation at Break (%)	Toughness (J)
BC	575.5 ± 151.3 ^a^	20.4 ± 7.1 ^c^	12.3 ± 0.4 ^d^	0.0103 ± 0.0020 ^h^
BC/PLA	319.9 ± 73.1 ^a^	22.7 ± 3.3 ^c^	18.7 ± 0.6 ^e^	0.0209 ± 0.0054 ^i^
BC/PLA/Lip	810.4 ± 58.5 ^b^	23.2 ± 2.0 ^c^	7.6 ± 1.1 ^f^	0.0063 ± 0.0005 ^j^

Different letters represents significantly different values (*p* < 0.1).
